# Bacteriological Profile of Patients With Stroke-Associated Pneumonia and Antimicrobial Susceptibility of Pathogens: A Cross-Sectional Study

**DOI:** 10.7759/cureus.74150

**Published:** 2024-11-21

**Authors:** Smrutisree Mohapatra, Basanti Kumari Pathi, Ipsa Mohapatra, Nipa Singh, Jyoti Prakash Sahoo, Narendra Kumar Das, Dipti Pattnaik

**Affiliations:** 1 Microbiology, Kalinga Institute of Medical Sciences, Bhubaneswar, IND; 2 Community Medicine, Kalinga Institute of Medical Sciences, Bhubaneswar, IND; 3 Pharmacology, Kalinga Institute of Medical Sciences, Bhubaneswar, IND; 4 Neurological Surgery, Kalinga Institute of Medical Sciences, Bhubaneswar, IND

**Keywords:** antibiotic susceptibility testing, bacterial drug resistance, cerebrovascular accident (stroke), culture and sensitivity, enterobacterales, hospital-acquired pneumonia, non-fermenter, stroke-associated pneumonia, ventilator-associated pneumonia, vitek 2 system

## Abstract

Background and objectives: Stroke-associated pneumonia (SAP) is the aftermath of aspiration of oropharyngeal secretions or stomach content. Mechanical ventilation and lowered immunity and consciousness facilitate the etiopathogenesis of SAP. Antibiotic prophylaxis and repeated culture and sensitivity testing dampen the drug susceptibility patterns of the pathogens. We accomplished this study to determine the bacteriological profile of patients with SAP and the antimicrobial susceptibility patterns of the pathogenic bacteria.

Methods: This cross-sectional study was executed from August 2022 to May 2024 at Kalinga Institute of Medical Sciences (KIMS), Bhubaneswar, India. We included adult patients who endured treatment in the neurosurgery intensive care unit (ICU) throughout the study period due to a stroke and developed pneumonia within 48 hours of admission. The endotracheal tube (ET) aspirate and bronchoalveolar lavage (BAL) fluid specimens collected from the eligible participants were analyzed. Enriched and selective media such as 5% sheep blood agar, chocolate agar, and MacConkey agar were used to culture pathogenic bacteria. The VITEK 2 system was used to identify isolates and assess antimicrobial susceptibility testing (AST). The pathogenic bacteria and their antimicrobial susceptibility patterns were gauged. We leveraged R software (version 4.4.1) for data analysis.

Results: Two hundred forty bacterial isolates were found in the 181 eligible patients. Forty-eight (26.52%) participants were females. The median age of the study population was 64.50 (58.74-70.24) years. Fifty-nine (32.60%) participants had two different isolates in their culture reports. We found the following non-fermenters: *Acinetobacter baumannii* (55, 22.92%), *Pseudomonas aeruginosa* (31, 12.92%), *Burkholderia cepacia* (6, 2.50%), and *Elizabethkingia meningoseptica* (4, 1.67%). *Klebsiella pneumoniae* (88, 36.67%) and *Escherichia coli* (15, 6.25%) were the most commonly noticed Enterobacterales. Other Enterobacterales were *Proteus mirabilis* (9, 3.75%), *Serratia marcescens* (8, 3.33%), *Klebsiella oxytoca* (3, 1.25%), *Enterobacter aerogenes* (1, 0.42%), *Providentia stuartii* (1, 0.42%), and *Enterobacter cloacae *complex (5, 2.08%). *Staphylococcus aureus* (14, 5.83%) was the only gram-positive cocci in our study population. The sensitivity of *A. baumannii* was maximum for minocycline. *P. aeruginosa* was highly sensitive to imipenem and completely resistant to tigecycline. Minocycline was the only effective drug against *E. meningoseptica*. Similarly, the Enterobacterales had the greatest sensitivity for tigecycline. All 14 specimens of *S. aureus* were sensitive to both vancomycin and linezolid. They were responsive to tigecycline as well.

Conclusion: The most common pathogenic bacteria in our study were *K. pneumoniae*, *A. baumannii*, *P. aeruginosa*, *E. coli*, and *S. aureus*. Enterobacterales were highly sensitive to tigecycline. *A. baumannii* and *E. meningoseptica* had maximum sensitivity for minocycline. All isolates of *S. aureus* were sensitive to both vancomycin and linezolid. We warrant further research with a larger sample size to investigate the bacteriological profile among other critically ill patients and their AST findings.

## Introduction

Nowadays, stroke-associated pneumonia (SAP) is the norm, affecting 14% of stroke patients [1,2]. It increases the odds of prolonged hospitalization, morbidity, and mortality [3]. It enormously impacts health economics and the global healthcare burden [1,4]. Pathogenesis of SAP is multifaceted. Aspiration of oropharyngeal secretions and gastric contents into the lungs as an aftermath of unconsciousness, sedatives, and dysphagia predisposes patients to SAP in the first few days following stroke [1,5]. Lowered immunity and existing respiratory tract infections (RTIs) hasten the development of SAP [6].

Acute brain injury prompts peripheral immune cells to traverse into the brain parenchyma, exacerbating neuroinflammation and neurovascular damage [7,8]. Neutrophils trigger brain damage by generating inflammatory cytokines and reactive oxygen and nitrogen species [7-9]. Monocyte deactivation and transient lymphopenia are hallmarks of peripheral cellular immune response inhibition and infection susceptibility [1,10]. These events disrupt the tracheal epithelium, leading to compromised pulmonary clearance [1,11]. Consequently, restricted airway passage and impeded clearance of pulmonary secretions foster pneumonia development [10,11].

*Klebsiella pneumoniae*, *Pseudomonas aeruginosa*, *Staphylococcus aureus*, and *Escherichia coli* continue to be regarded as major culprits in SAP [12,13]. This bacteriological profile of SAP is analogous to that of hospital-acquired pneumonia (HAP) and community-acquired pneumonia (CAP) [12]. *Acinetobacter baumannii* and *Burkholderia cepacia* are another couple of SAP pathogens [12,14]. Lately, incidences of SAP due to *Serratia marcescens*, *Proteus mirabilis*, and *Elizabethkingia meningoseptica* are rising [12,15].

Mechanical ventilation (MV) is a staple in healthcare institutions to safeguard proper air circulation in seriously ill patients in intensive care units (ICUs). However, mechanical strain from the MV might undermine the lower respiratory tract’s potential to flush out microorganisms. Hence, those on MV are more prone to developing SAP, a risk factor for SAP [16]. The best-known specimens for detecting pathogens are endotracheal tube (ET) aspirate and bronchoalveolar lavage (BAL) fluid [17]. Antibiotic prophylaxis and frequent antimicrobial sensitivity testing (AST) modify the drug susceptibility patterns of pathogenic bacteria [12,18]. We performed this study to determine the bacteriological profile of patients with SAP and the antimicrobial susceptibility patterns of the pathogenic bacteria.

## Materials and methods

In this cross-sectional study, we investigated the culture reports of patients with SAP for various pathogenic bacteria and their drug susceptibility patterns. The study was conducted at Kalinga Institute of Medical Sciences (KIMS), Bhubaneswar, India, from August 2022 to May 2024. We had ethical permission (KIIT/KIMS/IEC/950/2022) from the Institutional Ethics Committee, KIMS, before initiating the study. The research adhered to institutional standards, good clinical practices, good laboratory practices, and the Declaration of Helsinki.

Study participants

In this study, we included adult patients who were admitted to the neurosurgery ICU during the stipulated period with a clinical, radiological diagnosis of stroke and developed pneumonia after 48 hours of their ICU admission. We excluded patients aged less than 18 years, those with clinically diagnosed pneumonia when admitted to the ICU, and any other neurological diagnosis other than stroke. We also excluded those patients who died before collecting samples for antimicrobial susceptibility testing (AST).

Study procedure

Sample Collection

The sociodemographic (e.g., age, gender) and clinical (e.g., immediate cause of stroke, Glasgow Coma Scale (GCS) score, systolic blood pressure, and serum sodium at ICU admission) traits of eligible participants were recorded. A 22-inch, 14F suction catheter with a mucus extractor collected the ET aspirate without bronchoscopy. The catheter was carefully navigated around 25-26 cm into the tube. After gently aspirating without saline, the catheter was removed from the tube. A sterile syringe injected 3-4 ml of 0.9% saline to flush the mucus collector. The ET aspirate was obtained in a sterile jar with a secure screw-cap lid. The BAL fluid was obtained by administering 30 to 50 mL of normal saline through a fibrotic bronchoscope that was attached to the peripheral bronchiolar ramifications. The saline was immediately aspirated and transferred to a dry, clean, sterile container.

Transport of Specimens and Microbiological Processing

Respiratory samples were collected and processed immediately. The samples were preserved at -80^°^C to -20^°^C if processing was expected to take more than 1-2 hours. The inoculating loop was heated and allowed to cool. Then, it was placed flat on the liquid surface. The slide was numbered, and its loop was placed flat into the center. The specimen was spread thinly on a glass slide, dried fully, and heat-fixed by passing it through the flame three times before staining. A circle was traced around the smear for better visualization.

Culture of Specimen and Growth Interpretation

Specimens were cultured in the biosafety cabinet using rubber gloves, personal protection equipment (PPE), and surgical masks. Enriched and selective media such as 5% sheep blood agar, chocolate agar, and MacConkey agar were used to culture respiratory infections. Specimens were put into culture media in appropriate quantities. Standard techniques were used for running semi-quantitative cultures employing a calibrated loop method and a nichrome wire loop with 0.01 ml of fluid. After primary inoculation, plates were aerobically incubated in a 5% CO_2_ incubator overnight. The plates were scrutinized for bacterial growth after 24 and 48 hours of incubation. Colony counts of ≥10^5^ and ≥10^4^ colony-forming units per milliliter (CFU/mL) were deemed significant for ET aspirate and BAL fluid, respectively. Counts below these thresholds were considered inconsequential. Gram stain studies and colony morphology were performed on plates, and significant development was observed. Plate colony morphology was compared to Gram stain results from culture smears.

VITEK^®^ System

Enzymatic assays were used for preliminary identification, including catalase, coagulase, and oxidase. The VITEK 2 system served to identify isolates and assess antimicrobial susceptibility using the Clinical and Laboratory Standard Institute (CLSI) 2022 cut-off values [19]. The VITEK^®^ 2 system made use of a 64-well colorimetric reagent card. Gram-negative and gram-positive bacteria were identified using separate cards. The metabolic activity of substrates in the well was used to identify organisms. The organism's response pattern was compared to a database to identify it with high confidence.

AST

It functions based on micro-broth dilution. The antibacterial agents were diluted twice in the cards' wells. The organism suspension was introduced to the well. An antimicrobial agent's minimum inhibitory concentration (MIC) was defined as the maximum dilution inhibiting the organism's growth. Colonies were isolated from an agar plate after 18-24 hours and prepared with broth or saline solution. A calibrated photometric instrument (Densichek^®^, bioMérieux, France) regulated suspension turbidity to 0.5 McFarland. The AST inoculum was automatically injected by a filling tube into a 64-well colorimetric reagent card with a specified antibiotic concentration. Cards were incubated, and optical readings were obtained every 15 minutes to assess light transmission through the wells (including the growth control well). Validated software analyzed growth kinetics across every well and interpreted MIC results. Antimicrobial susceptibility was provided after the Advanced Expert System (AES) verified the final MIC result.

Statistical analysis

We used convenience sampling for this cross-sectional study. The Shapiro-Wilk test was deployed to confirm the normality of the data distribution. The continuous data were expressed with median and interquartile range (IQR). The frequency and proportion were displayed for the categorical data. We portrayed pathogenic bacteria's AST findings (i.e., sensitive, intermediate, or resistant) through chord diagrams. Version 4.4.1 of the R software (Vienna, Austria) was utilized for data computation [20]. A p-value of 0.05 or less was deemed statistically significant.

## Results

A total of 2575 patients were admitted to the neurosurgery ICU of our hospital during the study period. Of them, only 1029 (39.96%) had a stroke. Two hundred twelve subjects died within 24 hours of ICU admission. Out of the remaining 817 patients, 181 (22.15%) developed pneumonia after 48 hours of ICU admission. Those 181 patients diagnosed with SAP were analyzed in this cross-sectional study. Table 1 shows the sociodemographic and clinical traits of the study participants. Forty-eight (26.52%) participants were females. The median age of the study population was 64.50 (58.74-70.24) years. The median GCS score at admission was 6.0 (4.0-8.5). A total of 240 bacterial isolates were found in the 181 participants. Fifty-nine (32.60%) participants had two different isolates in their culture reports. *A. baumannii* (55, 22.92%), *P.* *aeruginosa* (31, 12.92%), *B. cepacia* (6, 2.50%), and *E. meningoseptica* (4, 1.67%) were the non-fermenters that we found during our investigation. *K. pneumoniae* (88, 36.67%) and *E. coli* (15, 6.25%) were the most commonly noticed Enterobacterales. Other Enterobacterales were *P. mirabilis* (9, 3.75%), *S. marcescens* (8, 3.33%), K. *oxytoca* (3, 1.25%), *Enterobacter aerogenes* (1, 0.42%), *Providentia stuartii* (1, 0.42%), and *Enterobacter cloacae *complex (5, 2.08%). *S. aureus* (14, 5.83%) was the only gram-positive cocci in our study population.

**Table 1:** Sociodemographic and clinical traits of the study population The median (IQR) was used to depict the continuous variables. n (%) was used to display the category values GCS: Glasgow Coma Scale; RTA: road traffic accident; IQR: interquartile range

Parameters	Value
Total participants	181
Age (years)	64.50 (58.74-70.24)
Female	48 (26.52%)
GCS score	6.0 (4.0-8.5)
Immediate cause of stroke	
RTA	104 (57.46%)
Non-RTA	77 (42.54%)
Systolic blood pressure (mm of Hg)	148.0 (142.0-164.0)
Serum sodium (mEq/L)	139.0 (133.0-143.5)
Comorbidities	
Type 2 diabetes mellitus	98 (54.14%)
Hypertension	117 (64.64%)
Bacterial isolates	240
Polymicrobial growth (in 181 patients)	59 (32.60%)
Monomicrobial growth (in 181 patients)	122 (67.40%)
Non-fermenters (out of 240 isolates)	96 (40.00%)
Acinetobacter baumannii	55 (22.92%)
Pseudomonas aeruginosa	31 (12.92%)
Burkholderia cepacia	6 (2.50%)
Elizabethkingia meningoseptica	4 (1.67%)
Enterobacterales (out of 240 isolates)	130 (54.17%)
Klebsiella pneumoniae	88 (36.67%)
Escherichia coli	15 (6.25%)
Proteus mirabilis	9 (3.75%)
Serratia marcescens	8 (3.33%)
*Enterobacter cloacae* complex	5 (2.08%)
Klebsiella oxytoca	3 (1.25%)
Enterobacter aerogenes	1 (0.42%)
Providentia stuartii	1 (0.42%)
Gram-positive cocci (out of 240 isolates)	14 (5.83%)
Staphylococcus aureus	14 (5.83%)

**Figure 1:**
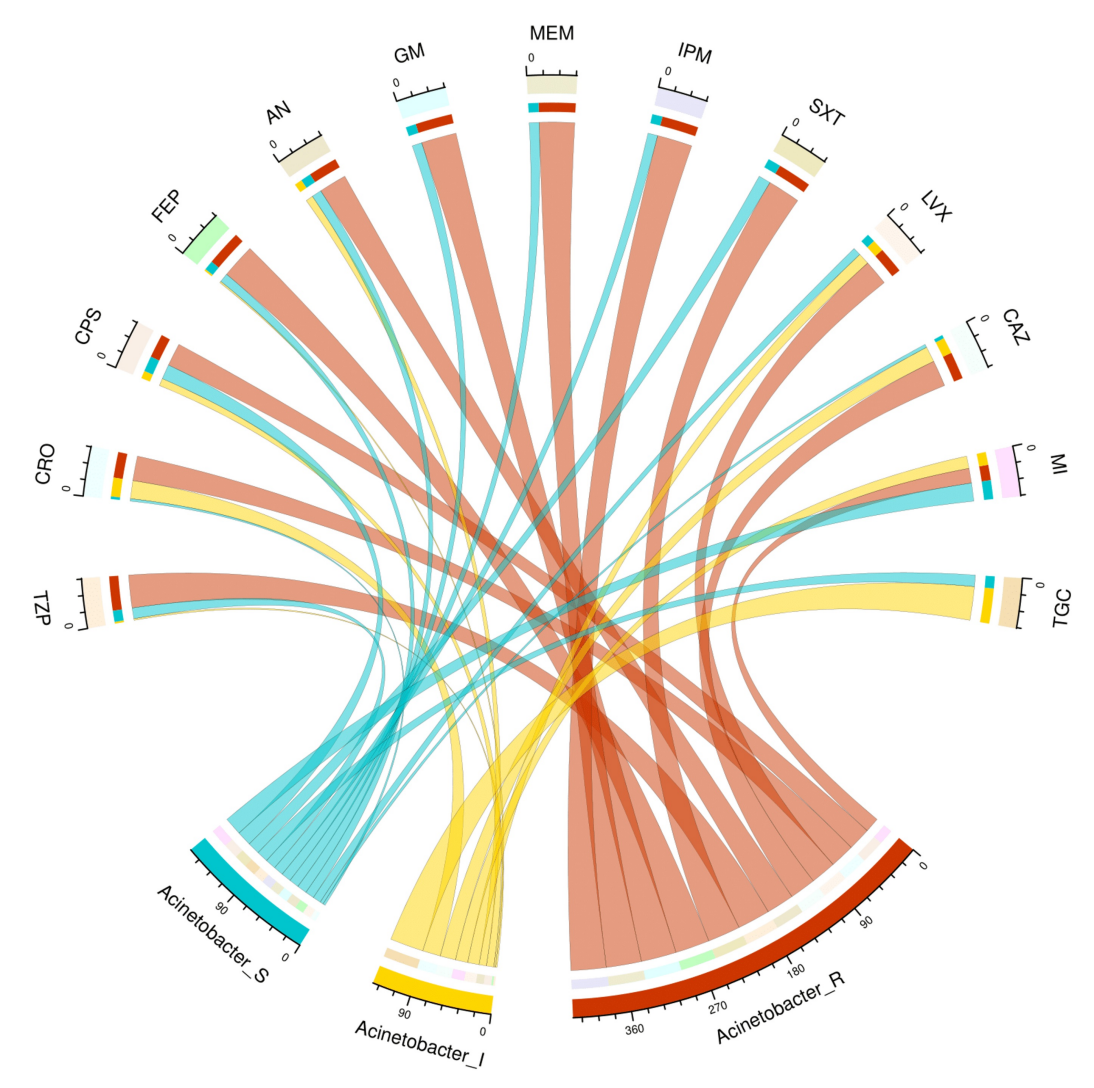
Antibiotic susceptibility pattern of Acinetobacter baumannii The lower portion of the plot portrays the antibiotic susceptibility patterns (S: sensitive, I: intermediate, and R: resistant) of *A. baumannii*, and the upper portion illustrates 13 antibiotics used in antimicrobial sensitivity testing. The bands between the lower and upper sections display all the findings for *A. baumannii*. The number of different types of antibiotic susceptibility of *A. baumannii* associated with the 13 drugs mentioned above correlates precisely with the bandwidths. TZP: piperacillin-tazobactam; CRO: ceftriaxone; CPS: cefoperazone-sulbactam; FEP: cefepime; AN: amikacin; GM: gentamicin; MEM: meropenem; IPM: imipenem; SXT: cotrimoxazole; LVX: levofloxacin; CAZ: ceftazidime; MI: minocycline; TGC: tigecycline

The chord diagram in Figure 1 showcases the antibiotic susceptibility pattern of 55 isolates of *A. baumannii* among the study participants. Minocycline was the most effective antibiotic for the bacterial isolates (22, 40%), followed by cefoperazone-sulbactam (18, 33%), cotrimoxazole (15, 27%), and tigecycline (14, 25%). These isolates showed incidences of resistance to all drugs except tigecycline. Table 2 narrates the frequency and proportion of the drug susceptibility pattern of *A. baumannii*.

**Table 2:** Antibiotic susceptibility patterns of non-fermenters Antibiotic susceptibility patterns (i.e., sensitive, intermediate, and resistant) of non-fermenters are depicted as frequency and proportions. TZP: piperacillin-tazobactam; CRO: ceftriaxone; CPS: cefoperazone-sulbactam; FEP: cefepime; AN: amikacin; GM: gentamicin; MEM: meropenem; IPM: imipenem; SXT: cotrimoxazole; LVX: levofloxacin; CAZ: ceftazidime; MI: minocycline; ATM: aztreonam; TGC: tigecycline; NA: not applicable

Bacteria	TZP	CRO	CPS	FEP	AN	GM	MEM	IPM	SXT	LVX	CAZ	MI	ATM	TGC
*Acinetobacter baumannii *(n = 55)														
Sensitive	13 (23%)	3 (5%)	18 (33%)	11 (20%)	12 (22%)	12 (22%)	12 (22%)	12 (22%)	15 (27%)	10 (18%)	4 (7%)	22 (40%)	NA	14 (25%)
Intermediate	2 (4%)	22 (40%)	9 (16%)	2 (4%)	9 (16%)	0	0	0	0	14 (25%)	19 (35%)	15 (27%)		41 (75%)
Resistant	40 (73%)	30 (55%)	28 (51%)	42 (76%)	34 (62%)	43 (78%)	43 (78%)	43 (78%)	40 (73%)	31 (56%)	32 (58%)	18 (33%)		0
*Burkholderia cepacia* (n = 6)														
Sensitive	2 (33%)	0	2 (33%)	2 (33%)	2 (33%)	2 (33%)	3 (50%)	2 (33%)	2 (33%)	2 (33%)	3 (50%)	1 (17%)	2 (33%)	0
Intermediate	1 (17%)	5 (83%)	3 (50%)	1 (17%)	1 (17%)	1 (17%)	1 (17%)	1 (17%)	1 (17%)	1 (17%)	2 (33%)	3 (50%)	2 (33%)	5 (83%)
Resistant	3 (50%)	1 (17%)	1 (17%)	3 (50%)	3 (50%)	3 (50%)	2 (33%)	3 (50%)	3 (50%)	3 (50%)	1 (17%)	2 (33%)	2 (33%)	1 (17%)
*Pseudomonas aeruginosa* (n = 31)														
Sensitive	17 (55%)	NA	19 (61%)	19 (61%)	19 (61%)	9 (29%)	17 (55%)	20 (65%)	NA	15 (48%)	17 (55%)	NA	12 (39%)	0
Intermediate	3 (10%)		2 (6%)	2 (6%)	1 (3%)	10 (32%)	2 (6%)	1 (3%)		3 (10%)	4 (13%)		8 (26%)	0
Resistant	11 (35%)		10 (32%)	10 (32%)	11 (35%)	12 (39%)	12 (39%)	10 (32%)		13 (42%)	10 (32%)		11 (35%)	31 (100%)
*Elizabethkingia meningoseptica* (n = 4)														
Sensitive	0	0	0	0	0	0	0	0	0	0	0	2 (50%)	0	NA
Intermediate	0	2 (50%)	0	0	0	1 (25%)	0	0	0	2 (50%)	2 (50%)	0	2 (50%)	
Resistant	4 (100%)	2 (50%)	4 (100%)	4 (100%)	4 (100%)	3 (75%)	4 (100%)	4 (100%)	4 (100%)	2 (50%)	2 (50%)	2 (50%)	2 (50%)	

**Figure 2:**
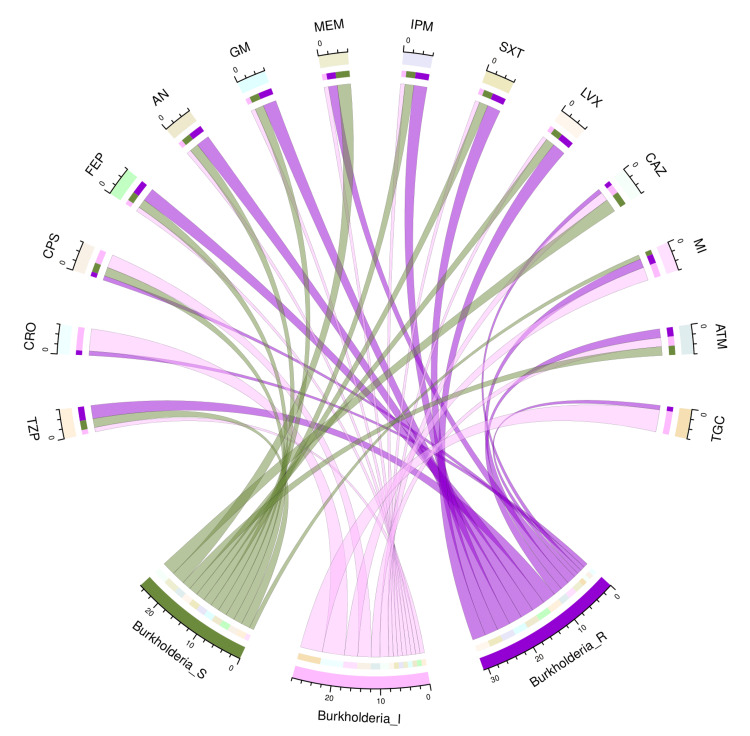
Antibiotic susceptibility pattern of Burkholderia cepacia The lower portion of the plot portrays the antibiotic susceptibility patterns (S: sensitive, I: intermediate, and R: resistant) of *B. cepacia*. The upper portion illustrates 14 antibiotics used in antimicrobial sensitivity testing. The bands between the lower and upper sections display all the findings for *B. cepacia*. As mentioned earlier, the number of different types of antibiotic susceptibility of *B. cepacia* associated with the 14 drugs correlates precisely with the bandwidths. TZP: piperacillin-tazobactam; CRO: ceftriaxone; CPS: cefoperazone-sulbactam; FEP: cefepime; AN: amikacin; GM: gentamicin; MEM: meropenem; IPM: imipenem; SXT: cotrimoxazole; LVX: levofloxacin; CAZ: ceftazidime; MI: minocycline; ATM: aztreonam; TGC: tigecycline

The chord diagram in Figure 2 showcases the antibiotic susceptibility pattern of six isolates of *B. cepacia* among the study participants. Three of those six bacterial isolates (50%) were sensitive to both meropenem and ceftazidime. None of the isolates were sensitive to ceftriaxone and tigecycline. Table 2 narrates the frequency and proportion of the drug susceptibility pattern of *B. cepacia*.

**Figure 3:**
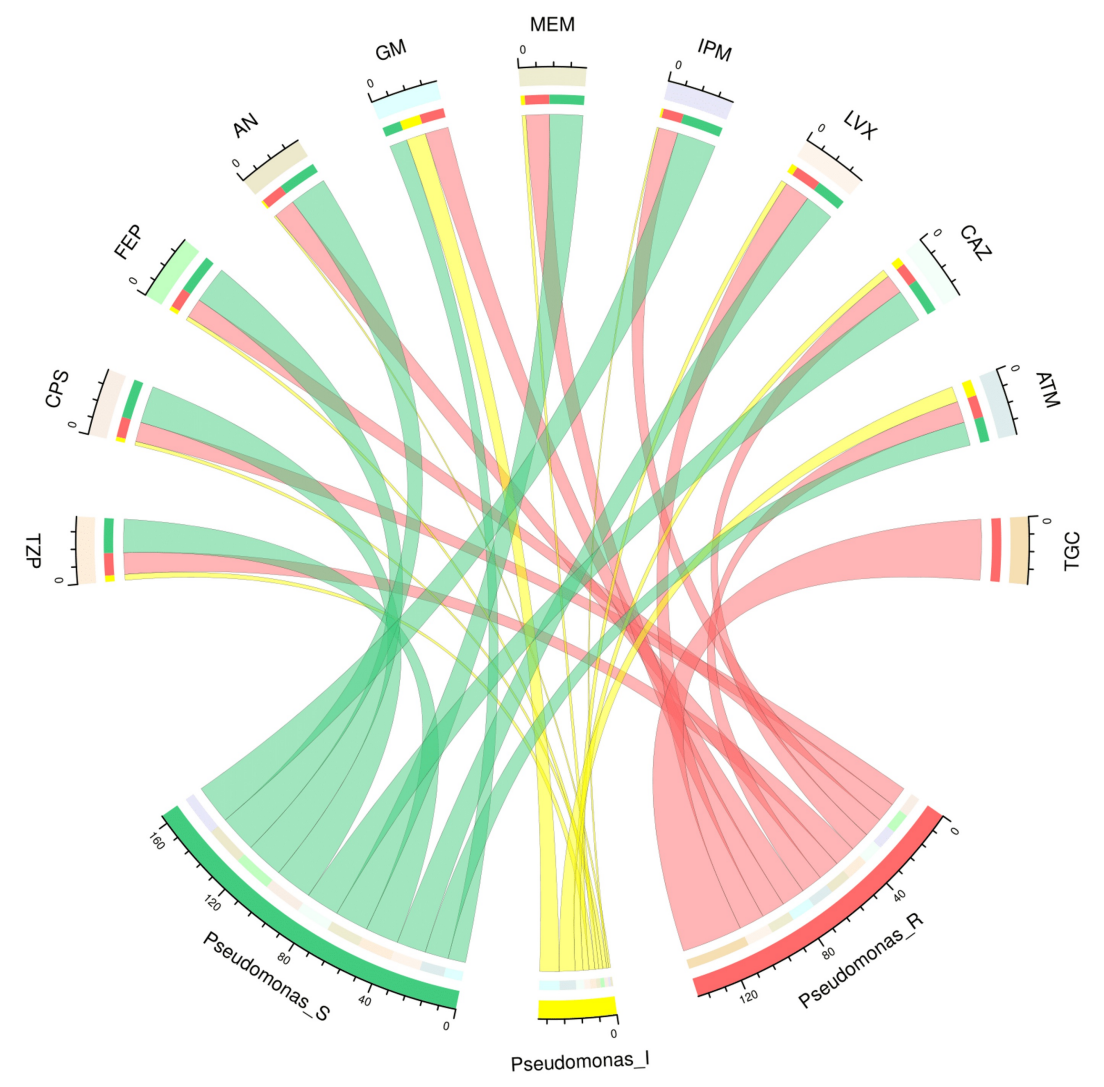
Antibiotic susceptibility pattern of Pseudomonas aeruginosa The lower portion of the plot portrays the antibiotic susceptibility patterns (S: sensitive, I: intermediate, and R: resistant) of *P. aeruginosa*, and the upper portion illustrates 11 antibiotics used in antimicrobial sensitivity testing. The bands between the lower and upper sections display all the findings for *P. aeruginosa*. As mentioned above, the number of different types of antibiotic susceptibility of *P. aeruginosa* associated with the 11 drugs correlates precisely with the bandwidths. TZP: piperacillin-tazobactam; CPS: cefoperazone-sulbactam; FEP: cefepime; AN: amikacin; GM: gentamicin; MEM: meropenem; IPM: imipenem; LVX: levofloxacin; CAZ: ceftazidime; ATM: aztreonam; TGC: tigecycline

The chord diagram in Figure 3 showcases the antibiotic susceptibility pattern of 31 isolates of *P. aeruginosa* among the study participants. The sensitivity of these bacterial isolates was maximum for imipenem (20, 65%), followed by cefoperazone-sulbactam (19, 61%), cefepime (19, 61%), amikacin (19, 61%), meropenem (17, 55%), and ceftazidime (17, 55%). All 31 isolates were resistant to tigecycline. Resistance against other drugs was found in nearly 1/3 of these isolates. Table 2 narrates the frequency and proportion of the drug susceptibility pattern of *P. aeruginosa*.

**Figure 4:**
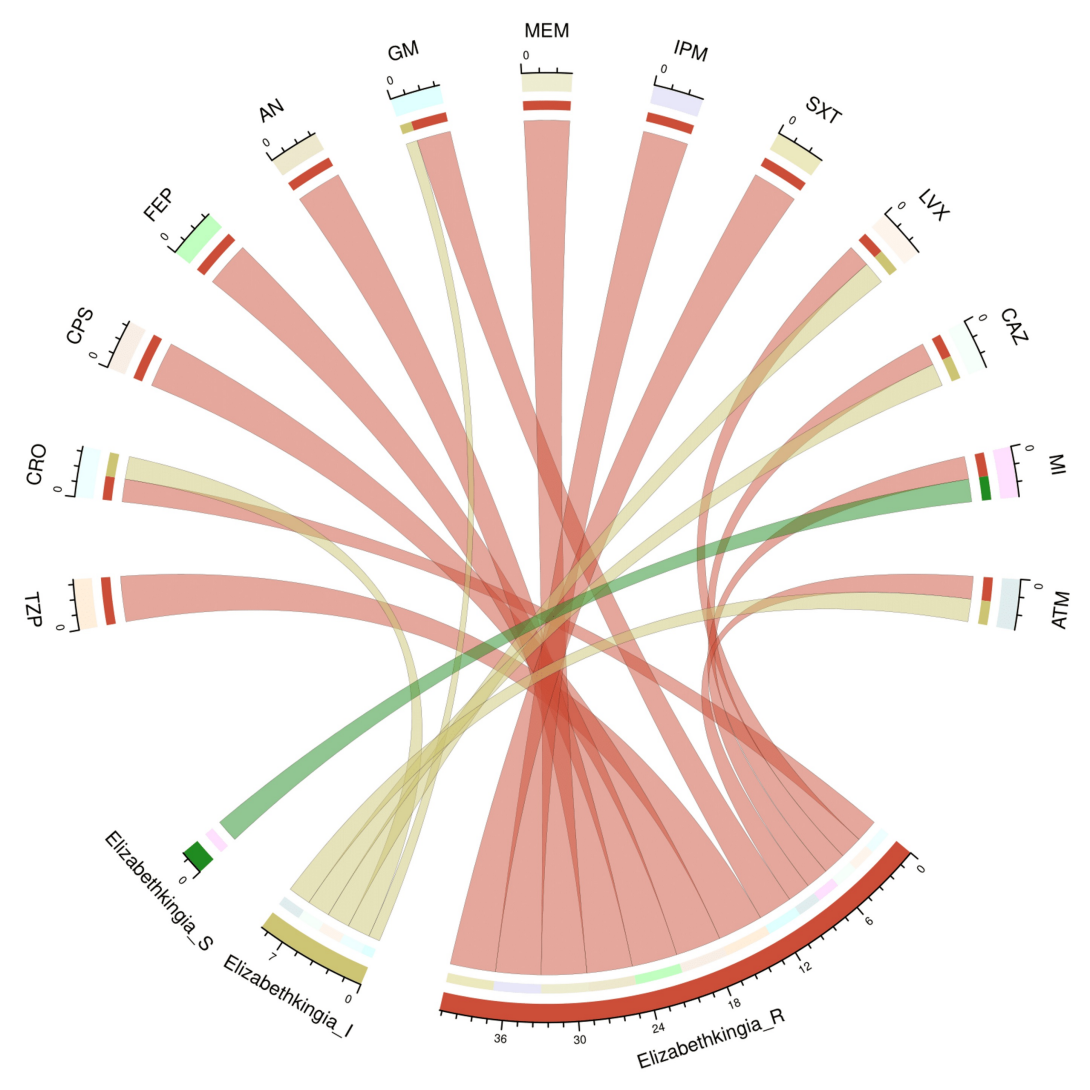
Antibiotic susceptibility pattern of Elizabethkingia meningoseptica The lower portion of the plot portrays the antibiotic susceptibility patterns (S: sensitive, I: intermediate, and R: resistant) of *E. meningoseptica*. The upper portion illustrates 13 antibiotics used in antimicrobial sensitivity testing. The bands between the lower and upper sections display all the findings for *E. meningoseptica*. The number of different types of antibiotic susceptibility of *E. meningoseptica* associated with the 13 drugs mentioned above correlates precisely with the bandwidths. TZP: piperacillin-tazobactam; CRO: ceftriaxone; CPS: cefoperazone-sulbactam; FEP: cefepime; AN: amikacin; GM: gentamicin; MEM: meropenem; IPM: imipenem; SXT: cotrimoxazole; LVX: levofloxacin; CAZ: ceftazidime; MI: minocycline; ATM: aztreonam

The chord diagram in Figure 4 showcases the antibiotic susceptibility pattern of four isolates of *E. meningoseptica* among the study participants. The bacterial isolates were sensitive only to minocycline (2, 50%). All four isolates showed resistance against piperacillin-tazobactam, cefoperazone-sulbactam, cefepime, amikacin, meropenem, imipenem, and cotrimoxazole. Table 2 narrates the frequency and proportion of the drug susceptibility pattern of *E. meningoseptica*.

**Figure 5:**
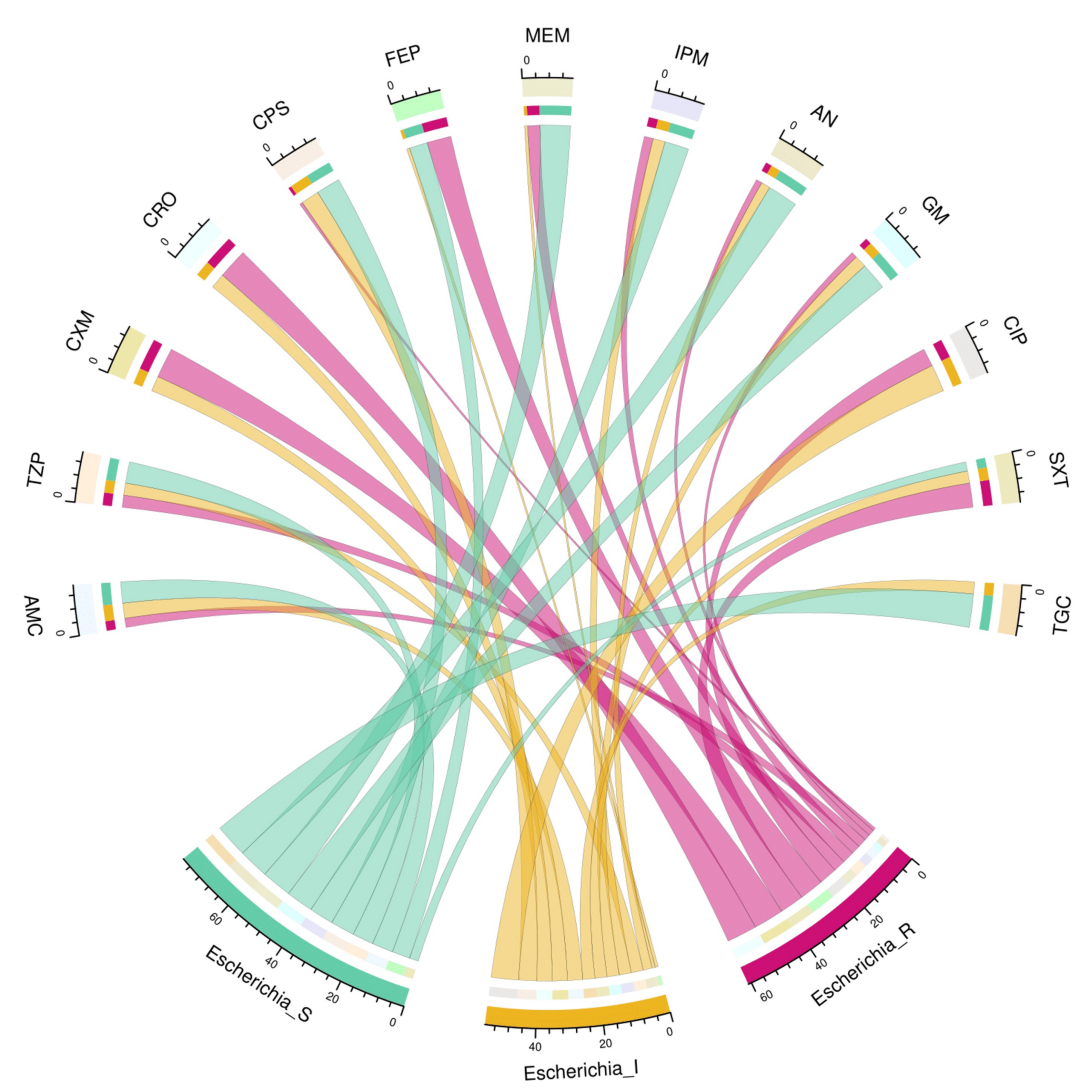
Antibiotic susceptibility pattern of Escherichia coli The lower portion of the plot portrays the antibiotic susceptibility patterns (S: sensitive, I: intermediate, and R: resistant) of *E. coli*, and the upper portion illustrates 13 antibiotics used in antimicrobial sensitivity testing. The bands between the lower and upper sections display all the findings for *E. coli*. As mentioned earlier, the number of different types of antibiotic susceptibility of *E. coli* associated with the 13 drugs correlates precisely with the bandwidths. AMC: amoxicillin-clavulanic acid; TZP: piperacillin-tazobactam; CXM: cefuroxime; CRO: ceftriaxone; CPS: cefoperazone-sulbactam; FEP: cefepime; MEM: meropenem; IPM: imipenem; AN: amikacin; GM: gentamicin; CIP: ciprofloxacin; SXT: cotrimoxazole; TGC: tigecycline

The chord diagram in Figure 5 showcases the antibiotic susceptibility pattern of 15 *E. coli* isolates among the study participants. The bacterial isolates were maximally sensitive to tigecycline (11, 73%), followed by meropenem (10, 67%), amikacin (10, 67%), and gentamicin (9, 60%). All 15 isolates showed resistance against cefuroxime, ceftriaxone, and ciprofloxacin. None of these isolates demonstrated resistance against tigecycline. Table 3 narrates the frequency and proportion of the drug susceptibility pattern of *E. coli*.

**Table 3:** Antibiotic susceptibility patterns of Enterobacterales Antibiotic susceptibility patterns (i.e., sensitive, intermediate, and resistant) of Enterobacterales are depicted as frequency and proportions. AMC: amoxicillin-clavulanic acid; TZP: piperacillin-tazobactam; CXM: cefuroxime; CRO: ceftriaxone; CPS: cefoperazone-sulbactam; FEP: cefepime; MEM: meropenem; IPM: imipenem; AN: amikacin; GM: gentamicin; CIP: ciprofloxacin; SXT: cotrimoxazole; TGC: tigecycline

Bacteria	AMC	TZP	CXM	CRO	CPS	FEP	MEM	IPM	AN	GM	CIP	SXT	TGC
Escherichia coli (n = 15)													
Sensitive	7 (47%)	7 (47%)	0	0	8 (53%)	6 (40%)	10 (67%)	8 (53%)	10 (67%)	9 (60%)	0	3 (20%)	11 (73%)
Intermediate	5 (3%)	4 (27%)	5 (33%)	5 (33%)	6 (40%)	1 (7%)	1 (7%)	4 (27%)	3 (20%)	4 (27%)	9 (60%)	4 (27%)	4 (27%)
Resistant	3 (20%)	4 (27%)	10 (67%)	10 (67%)	1 (7%)	8 (53%)	4 (27%)	3 (20%)	2 (13%)	2 (13%)	6 (40%)	8 (53%)	0
Klebsiella pneumoniae (n = 88)													
Sensitive	7 (8%)	8 (9%)	3 (3%)	3 (3%)	9 (10%)	6 (7%)	14 (16%)	8 (9%)	16 (18%)	32 (36%)	5 (6%)	16 (18%)	52 (59%)
Intermediate	22 (25%)	20 (23%)	22 (25%)	21 (24%)	19 (22%)	2 (2%)	1 (1%)	26 (30%)	33 (38%)	21 (24%)	19 (22%)	21 (24%)	19 (22%)
Resistant	59 (67%)	60 (68%)	63 (72%)	64 (73%)	60 (68%)	80 (91%)	73 (83%)	54 (61%)	39 (44%)	35 (40%)	64 (73%)	51 (58%)	17 (19%)
Miscellaneous bacteria (n = 27)													
Sensitive	4 (15%)	16 (59%)	2 (7%)	4 (15%)	9 (33%)	12 (44%)	8 (30%)	7 (26%)	11 (41%)	10 (37%)	6 (22%)	13 (48%)	13 (48%)
Intermediate	13 (48%)	5 (19%)	5 (19%)	7 (26%)	7 (26%)	0	4 (15%)	5 (19%)	3 (11%)	3 (11%)	1 (4%)	4 (15%)	6 (22%)
Resistant	10 (37%)	6 (22%)	20 (74%)	16 (59%)	11 (41%)	15 (56%)	15 (56%)	15 (56%)	13 (48%)	14 (52%)	20 (74%)	10 (37%)	8 (30%)

**Figure 6:**
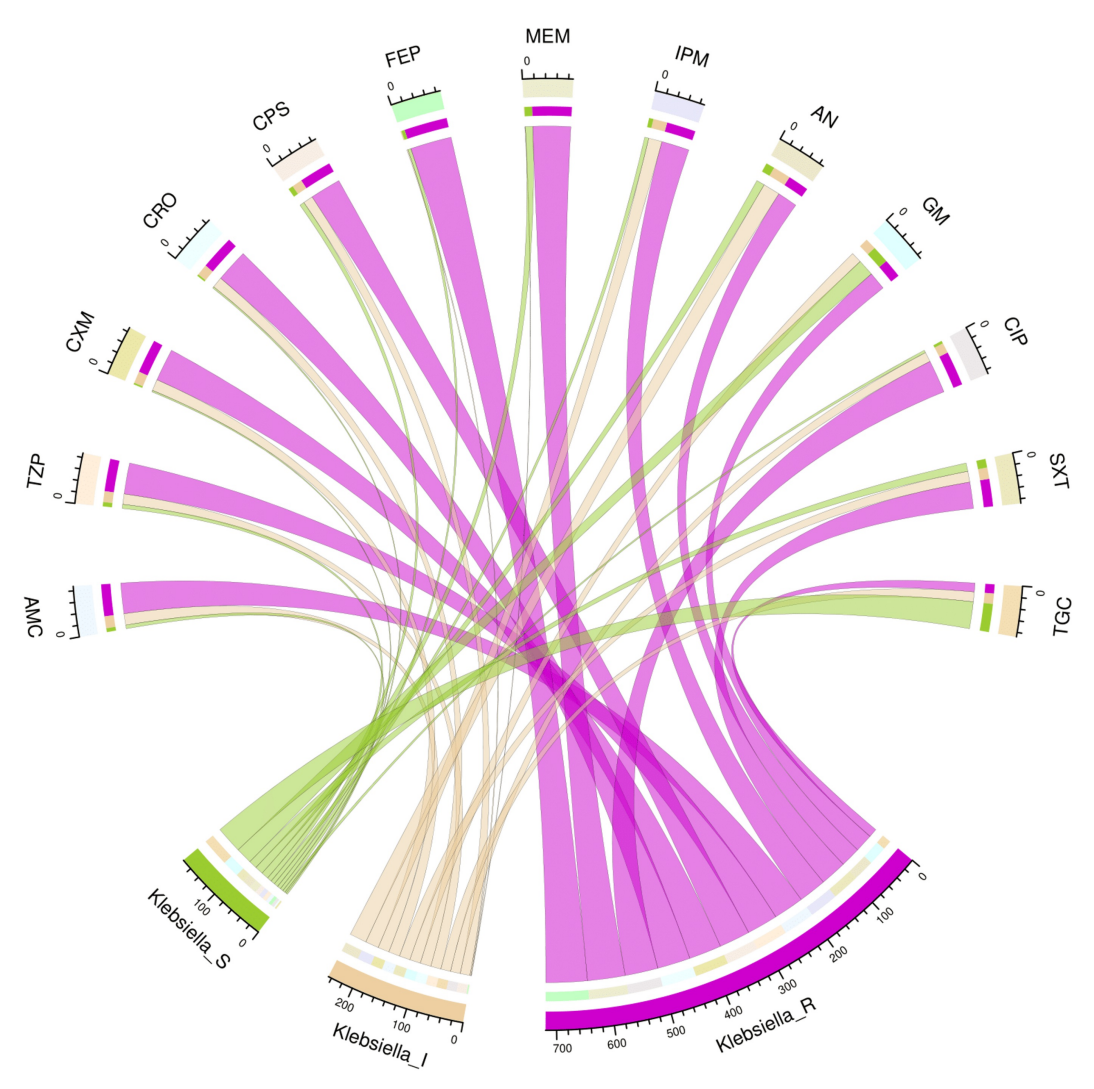
Antibiotic susceptibility pattern of Klebsiella pneumoniae The lower portion of the plot portrays the antibiotic susceptibility patterns (S: sensitive, I: intermediate, and R: resistant) of *K. pneumoniae*, and the upper portion illustrates 13 antibiotics used in antimicrobial sensitivity testing. The bands between the lower and upper sections display all the findings for *K. pneumoniae*. The number of different types of antibiotic susceptibility of *K. pneumoniae* associated with the 13 drugs, as mentioned above, correlates precisely with the bandwidths. AMC: amoxicillin-clavulanic acid; TZP: piperacillin-tazobactam; CXM: cefuroxime; CRO: ceftriaxone; CPS: cefoperazone-sulbactam; FEP: cefepime; MEM: meropenem; IPM: imipenem; AN: amikacin; GM: gentamicin; CIP: ciprofloxacin; SXT: cotrimoxazole; TGC: tigecycline

The chord diagram in Figure 6 showcases the antibiotic susceptibility pattern of 88 isolates of *K. pneumoniae* among the study participants. The bacterial isolates were maximally sensitive to tigecycline (52, 59%), followed by gentamicin (32, 36%), amikacin (16, 18%), cotrimoxazole (16, 18%), and meropenem (14, 16%). The majority of these isolates were resistant to almost all drugs. Table 3 narrates the frequency and proportion of the drug susceptibility pattern of *K. pneumoniae*.

**Figure 7:**
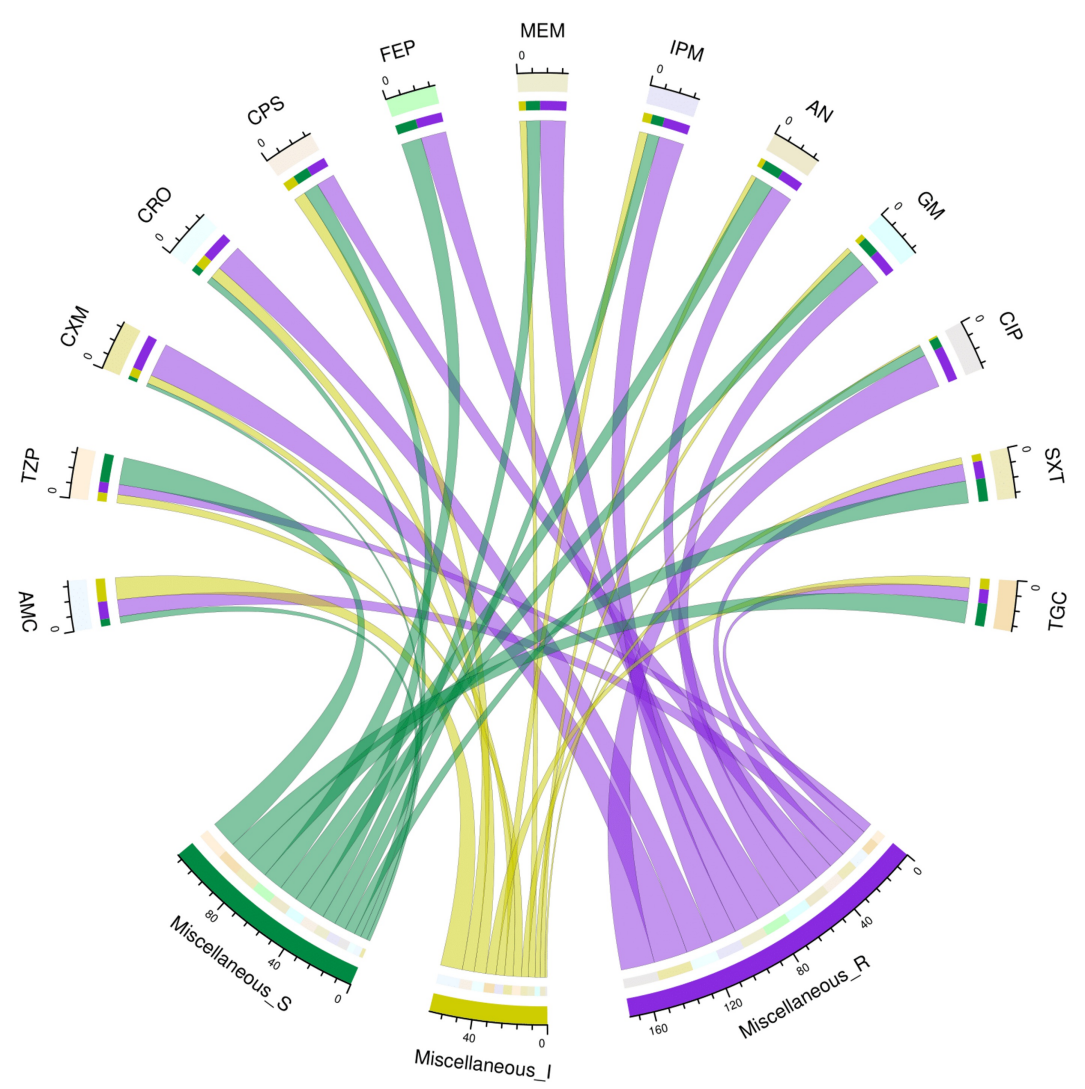
Antibiotic susceptibility pattern of miscellaneous Enterobacterales The lower portion of the plot portrays the antibiotic susceptibility patterns (S: sensitive, I: intermediate, and R: resistant) of miscellaneous Enterobacterales, and the upper portion illustrates 13 antibiotics used in antimicrobial sensitivity testing. The miscellaneous Enterobacterales include the following bacteria: *Klebsiella oxytoca*, *Serratia marcescens*, *Enterobacter aerogenes*, *Proteus mirabilis*, *Providentia stuartii*, and *Enterobacter cloacae *complex. The bands between the lower and upper sections display all the findings for miscellaneous Enterobacterales. The number of different types of antibiotic susceptibility of miscellaneous Enterobacterales associated with the 13 drugs mentioned above correlates precisely with the bandwidths. AMC: amoxicillin-clavulanic acid; TZP: piperacillin-tazobactam; CXM: cefuroxime; CRO: ceftriaxone; CPS: cefoperazone-sulbactam; FEP: cefepime; MEM: meropenem; IPM: imipenem; AN: amikacin; GM: gentamicin; CIP: ciprofloxacin; SXT: cotrimoxazole; TGC: tigecycline

The chord diagram in Figure 7 showcases the antibiotic susceptibility pattern of 27 isolates of lesser-known or miscellaneous Enterobacterales among the study participants. They were *K. oxytoca* (n = 3), *S. marcescens* (n = 8), *E. aerogenes* (n = 1), *P. mirabilis* (n = 9), *P. stuartii* (n = 1), and *E. cloacae complex* (n = 5). Collectively, these bacterial isolates were maximally sensitive to piperacillin-tazobactam (16, 59%), followed by cotrimoxazole (13, 48%), tigecycline (13, 48%), and amikacin (11, 41%). Twenty (74%) of these isolates showed resistance against cefuroxime and ciprofloxacin. Tables 3-4 narrate the frequency and proportion of the drug susceptibility pattern of miscellaneous Enterobacterales.

**Table 4:** Antibiotic susceptibility patterns of miscellaneous Enterobacterales Antibiotic susceptibility patterns (i.e., sensitive, intermediate, and resistant) of miscellaneous bacteria (belonging to the Enterobacterales group mentioned in the previous table) are depicted as frequency and proportions. AMC: amoxicillin-clavulanic acid; TZP: piperacillin-tazobactam; CXM: cefuroxime; CRO: ceftriaxone; CPS: cefoperazone-sulbactam; FEP: cefepime; MEM: meropenem; IPM: imipenem; AN: amikacin; GM: gentamicin; CIP: ciprofloxacin; SXT: cotrimoxazole; TGC: tigecycline

Bacteria	AMC	TZP	CXM	CRO	CPS	FEP	MEM	IPM	AN	GM	CIP	SXT	TGC
*Klebsiella oxytoca *(n = 3)													
Sensitive	2 (67%)	2 (67%)	1 (33%)	1 (33%)	2 (67%)	3 (100%)	2 (67%)	2 (67%)	1 (33%)	2 (67%)	1 (33%)	2 (67%)	2 (67%)
Intermediate	1 (33%)	1 (33%)	2 (67%)	2 (67%)	1 (33%)	0	1 (33%)	1 (33%)	1 (33%)	1 (33%)	1 (33%)	1 (33%)	1 (33%)
Resistant	0	0	0	0	0	0	0	0	1 (33%)	0	1 (33%)	0	0
*Serratia marcescens* (n = 8)													
Sensitive	1 (12%)	4 (50%)	1 (12%)	1 (12%)	4 (50%)	3 (38%)	2 (25%)	2 (25%)	4 (50%)	3 (38%)	2 (25%)	3 (38%)	3 (38%)
Intermediate	4 (50%)	2 (25%)	1 (12%)	2 (25%)	3 (38%)	0	2 (25%)	2 (25%)	1 (12%)	0	0	2 (25%)	2 (25%)
Resistant	3 (38%)	2 (25%)	6 (75%)	5 (62%)	1 (12%)	5 (62%)	4 (50%)	4 (50%)	3 (38%)	5 (62%)	6 (75%)	3 (38%)	3 (38%)
*Enterobacter aerogenes* (n = 1)													
Sensitive	1 (100%)	1 (100%)	0	0	1 (100%)	1 (100%)	1 (100%)	1 (100%)	1 (100%)	1 (100%)	1 (100%)	1 (100%)	1 (100%)
Intermediate	0	0	0	1 (100%)	0	0	0	0	0	0	0	0	0
Resistant	0	0	1 (100%)	0(0%)	0	0	0	0	0	0	0	0	0
*Proteus mirabilis* (n = 9)													
Sensitive	0	5 (56%)	0	0	0	1 (11%)	0	0	0	1 (11%)	0	3 (33%)	3 (33%)
Intermediate	4 (44%)	1 (11%)	0	0	0	0	0	1 (11%)	0	1 (11%)	0	0	1 (11%)
Resistant	5 (56%)	3 (33%)	9 (100%)	9 (100%)	9 (100%)	8 (89%)	9 (100%)	8 (89%)	9 (100%)	7 (78%)	9 (100%)	6 (67%)	5 (56%)
*Providentia stuartii *(n = 1)													
Sensitive	0	0	0	0	0	1 (100%)	0	0	1 (100%)	0	0	1 (100%)	1 (100%)
Intermediate	0	0	0	0	1 (100%)	0	0	0	0	0	0	0	0
Resistant	1 (100%)	1 (100%)	1 (100%)	1 (100%)	0	0	1 (100%)	1 (100%)	0	1 (100%)	1 (100%)	0	0
*Enterobacter cloacae* complex (n = 5)													
Sensitive	0	4 (80%)	0	2 (40%)	2 (40%)	3 (60%)	3 (60%)	2 (40%)	4 (80%)	3 (60%)	2 (40%)	3 (60%)	3 (60%)
Intermediate	4 (80%)	1 (20%)	2 (40%)	2 (40%)	2 (40%)	0	1 (20%)	1 (20%)	1 (20%)	1 (20%)	0	1 (20%)	2 (40%)
Resistant	1 (20%)	0	3 (60%)	1 (20%)	1 (20%)	2 (40%)	1 (20%)	2 (40%)	0	1 (20%)	3 (60%)	1 (20%)	0

**Figure 8:**
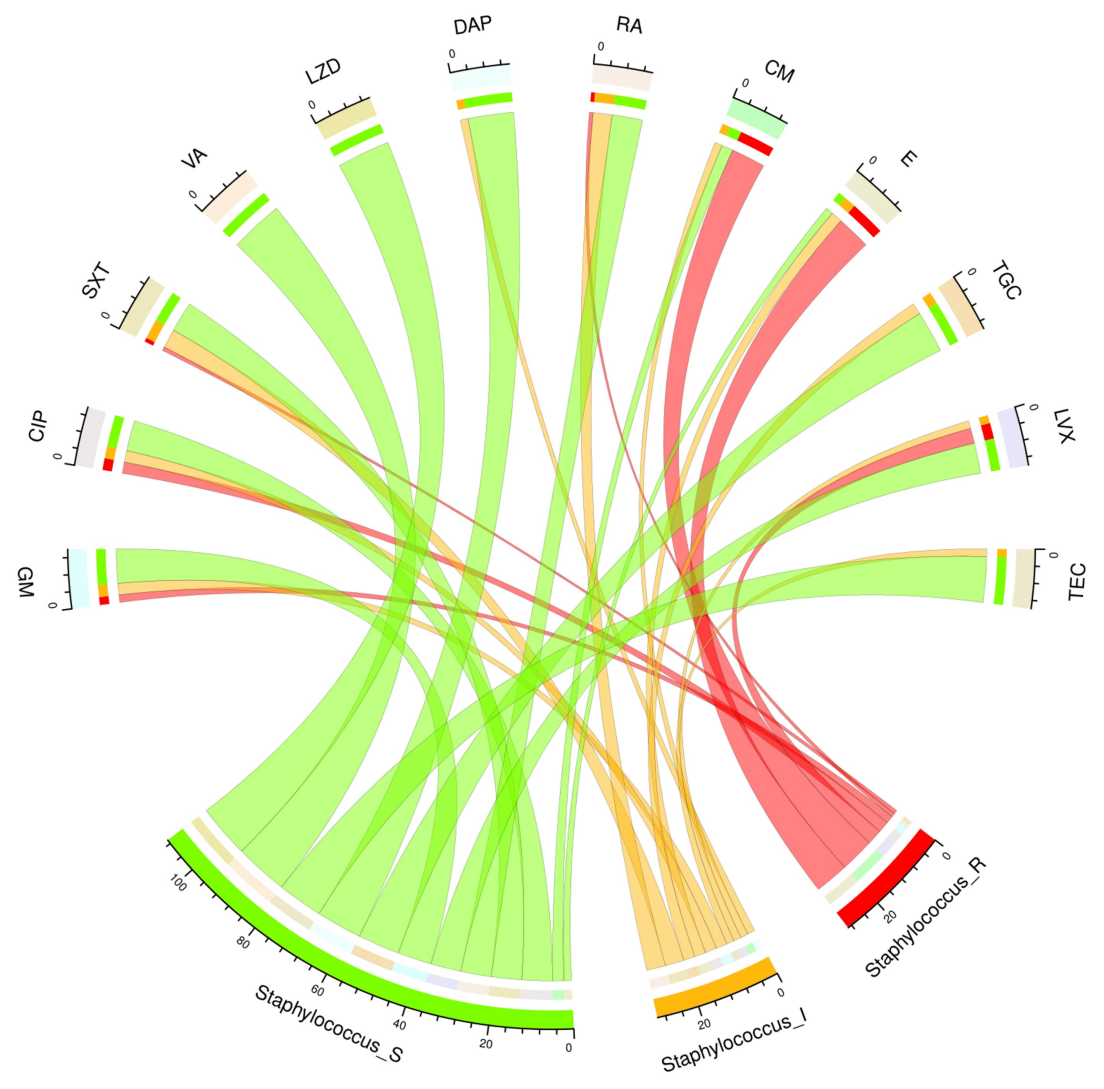
Antibiotic susceptibility pattern of Staphylococcus aureus The lower portion of the plot portrays the antibiotic susceptibility patterns (S: sensitive, I: intermediate, and R: resistant) of *S. aureus*, and the upper portion illustrates 12 antibiotics used in antimicrobial sensitivity testing. The bands between the lower and upper sections display all the findings for *S. aureus*. As mentioned earlier, the number of different types of antibiotic susceptibility of *S. aureus* associated with the 12 drugs correlates precisely with the bandwidths. GM: gentamicin; CIP: ciprofloxacin; SXT: cotrimoxazole; VA: vancomycin; LZD: linezolid; DAP: daptomycin; RA: rifampicin; CM: clindamycin; E: erythromycin; TGC: tigecycline; LVX: levofloxacin; TEC: teicoplanin

The chord diagram in Figure 8 showcases the antibiotic susceptibility pattern of 14 *S. aureus* isolates among the study participants. All 14 bacterial isolates were sensitive to vancomycin and linezolid. The other sensitive drugs were daptomycin (12, 86%), teicoplanin (12, 86%), and tigecycline (11, 79%). Nine (64%) of 14 isolates showed resistance against clindamycin and erythromycin. Table 5 narrates the frequency and proportion of the drug susceptibility pattern of *S. aureus*.

**Table 5:** Antibiotic susceptibility pattern of Gram-positive cocci Antibiotic susceptibility patterns (i.e., sensitive, intermediate, and resistant) of Gram-positive cocci are depicted as frequency and proportions. GM: gentamicin; CIP: ciprofloxacin; SXT: cotrimoxazole; VA: vancomycin; LZD: linezolid; DAP: daptomycin; RA: rifampicin; CM: clindamycin; E: erythromycin; TGC: tigecycline; LVX: levofloxacin; TEC: teicoplanin

Bacteria	GM	CIP	SXT	VA	LZD	DAP	RA	CM	E	TGC	LVX	TEC
*Staphylococcus aureus* (n = 14)												
Sensitive	9 (64%)	8 (57%)	8 (57%)	14 (100%)	14 (100%)	12 (86%)	8 (57%)	3 (21%)	2 (14%)	11 (79%)	8 (57%)	12 (86%)
Intermediate	3 (21%)	3 (21%)	5 (36%)	0	0	2 (14%)	5 (36%)	2 (14%)	3 (21%)	3 (21%)	2 (14%)	2 (14%)
Resistant	2 (14%)	3 (21%)	1 (7%)	0	0	0	1 (7%)	9 (64%)	9 (64%)	0	4 (29%)	0

## Discussion

In this cross-sectional study, 181 (22.15%) of 817 patients admitted to the neurosurgery ICU with stroke developed pneumonia after 48 hours of admission. The study population's median age was 64.50 (58.74-70.24) years. The median GCS score at admission was 6.0 (4.0-8.5). There were 240 bacteria detected among those 181 individuals. In their culture reports, 59 (32.60%) subjects had two distinct isolates. The culture reports discovered non-fermenters (e.g., *A. baumannii*, *P. aeruginosa*, *B. cepacia*, and *E. meningoseptica*), Enterobacterales (e.g., *K. pneumoniae* and *E. coli*), and gram-positive cocci (e.g., *S. aureus*) in the ET aspirate and BAL fluid of the study participants. Our observations concorded with the findings of studies by Xu et al. [13], Aboulfotooh et al. [14], and Assefa et al. [18].

The majority of our study subjects were elderly individuals with type 2 diabetes mellitus and hypertension. More than half of them had strokes because of RTA. Elderly hypertensive males are more prone to suffering from transient ischemic attack (TIA) or stroke [21-23]. Lowered consciousness, mobility, and slower mucociliary clearance of airways favor the pathogens to dwell inside the pulmonary circuits [11,24,25]. The most common pathogens noticed among the 240 bacterial isolates were *K. pneumoniae*, *A. baumannii*, *P. aeruginosa*, *E. coli*, and *S. aureus*. We also detected newly emerging pathogenic bacteria causing pneumonia (i.e., *E. meningoseptica*, *P. mirabilis*, and *S. marcescens*).

*A. baumannii* exhibited the highest sensitivity to minocycline, followed by tigecycline. *B. cepacia* demonstrated maximum sensitivity for meropenem. However, tigecycline was ineffective for this pathogen. *P. aeruginosa* was extremely susceptible to imipenem but fully resistant to tigecycline. Lately, Feng et al. [26] revealed the sensitivity of *E. meningoseptica* toward many antibiotics. The study by Wu et al. [27] advocated that *E. meningoseptica* was completely susceptible to minocycline and colistin. In our study, minocycline was the sole medicine that effectively combated *E. meningoseptica*. It was resistant to all other antimicrobials. *K. pneumoniae* and *E. coli* were most sensitive to tigecycline and meropenem. *P. mirabilis* and *S. marcescens* exhibited maximum sensitivity for piperacillin-tazobactam. Nonetheless, *P. mirabilis* showed resistance against numerous antibiotics. Vancomycin and linezolid were effective against all *S. aureus* isolates. They also responded nicely to tigecycline, daptomycin, and teicoplanin.

The main strength of this study was data presentation through chord diagrams. Our study had some limitations as well. First, we should have analyzed the history of recent infections and antibiotic administration. Second, we did not investigate the correlation between AST findings, hospital stay, and fate. Third, pneumonia in ICU patients has multiple etiologies and risk factors. We did not assess any risk factors for the development of SAP.

## Conclusions

*K. pneumoniae*, *A. baumannii*, *P. aeruginosa*, *E. coli*, and *S. aureus* were the most prevalent pathogenic bacteria identified in our investigation. In addition, we found four cases of *E. meningoseptica*. Enterobacterales were extremely sensitive to tigecycline. Minocycline sensitivity was highest in *A. baumannii* and *E. meningoseptica* bacteria. All *S. aureus* isolates were susceptible to vancomycin and linezolid. We recommend more research with larger samples to gaze at the bacteriological profile of more critically ill individuals and their AST results.
